# Analysis of the Genetic Relationship and Inbreeding Coefficient of the Hetian Qing Donkey through a Simplified Genome Sequencing Technology

**DOI:** 10.3390/genes15050570

**Published:** 2024-04-28

**Authors:** Bo Liu, Shujuan Gong, Hanikezi Tulafu, Rongyin Zhang, Weikun Tao, Abulikemu Adili, Li Liu, Weiwei Wu, Juncheng Huang

**Affiliations:** 1 College of Animal Science and Technology, Northwest A&F University, Yangling 712100, China; liubogong@foxmail.com (B.L.); gongshujuan123@foxmail.com (S.G.); 2 Key Laboratory of Genetics Breeding and Reproduction of Xinjiang Wool Sheep & Cashmere Goat, Institute of Animal Science, Xinjiang Academy of Animal Sciences, Urumqi 830000, China; hnkz2023@foxmail.com (H.T.); taoweikun@hotmail.com (W.T.); 3 Research Institute of Animal Husbandry Quality Standards, Xinjiang Academy of Animal Husbandry Sciences, Urumqi 830000, China; huaer901@163.com; 4 Hetian District Animal Husbandry Technology Promotion Station, Hetian 848000, China; ablikimadil@126.com (A.A.); xjliul@163.com (L.L.); 5 Institute of Animal Husbandry, Xinjiang Academy of Animal Husbandry Sciences, Urumqi 830000, China

**Keywords:** Hetian Qing donkey, simplified genome, genetic diversity, population structure

## Abstract

The Hetian Qing donkey is an excellent local donkey breed in Xinjiang. It is of great significance to accelerate breeding and the speed of breeding and rejuvenation, as well as to understand the genetic basis of the strategies and population. This study collected a total of 4 male donkeys and 28 female donkeys. It then obtained genotype data through Simplified Genomic Sequencing (GBS) technology for data analysis. The results detected a total of 55,399 SNP loci, and the genotype detection rate of individuals was ≥90%. A total of 45,557 SNP loci were identified through quality control, of which 95.5% were polymorphic. The average minimum allele frequency was 0.250. The average observed heterozygosity was 0.347. The average expected heterozygosity was 0.340. The average IBS (state homologous) genetic distance was 0.268. ROH: 49 (homozygous fragments), with 73.47% of the length between 1 and 5 Mb. The average per-strip ROH length was 1.75 Mb. The mean inbreeding coefficient was 0.003. The 32 Hetian green donkeys could be divided into six families. The number of individuals in each family is significant. To sum up, the Hetian Qing donkey population has low heterozygosity, few families, and large differences in the number of individuals in each family, which can easily cause a loss of genetic diversity. In the subsequent process of seed protection, seed selection should be conducted according to the divided pedigree to ensure the long-term protection of the genetic resources of Hetian green donkeys.

## 1. Introduction

The Hetian Qing donkey (formerly known as the Gola donkey) is a valuable local medium-sized donkey breed in China. In 2009, the Hetian Qing donkey was listed in the national and local livestock and poultry genetic resources protection list by the state and Xinjiang Uygur Autonomous Region. Hetian Qing donkeys are mainly distributed in Qiaoda, Sangzhu, Muji, Zanggui, Piyaman townships, and other towns in the Hetian Pishan County. The main production area is in the Qiaoda township plain area, which has a typical continental warm tropical–arid desertification climate. It is rich in light and heat resources. The annual average temperature is 11.8 °C, the annual average precipitation is 51.3 mm, and the annual evaporation is 2700 mm [1]. Therefore, the Hetian Qing donkey favors a dry and warm climate and has strong disease resistance, resistance to rough feeding, resistance to hunger and thirst, early sexual maturity, high reproductive rate, fast growth speed, high physical performance and health, high meat production, leathery skin, and dual-use [2]. At the end of 2009, only approximately 2000 animals were in the county, which need to be protected urgently [3]. Therefore, strengthening the breeding of Hetian Qing donkey strains, carrying out seed selection and mating, and successful purification, rejuvenation, population expansion, and reproduction have become the primary tasks in Hetian Qing donkey species conservation. Male and female donkeys become sexually active around the age of one. Female donkeys can start breeding at this point under good dietary conditions. Female donkeys can be used for breeding up to 15–20 years old. The average gestation period of female donkeys is 361 days, the sexual maturity is 10–18 months, and 15 to 20 foals are born in their lifetimes. The survival rate of foals reaches more than 90% [4]. It is difficult to select and breed seeds and slow population expansion because of the species’ long breeding cycle and poor breeding technology.

In recent years, with the continuous progress in molecular biological technology and substantial reductions in the cost of mutation detection, obtaining a large amount of SNP information is easier. High-flux mutation detection technologies such as genome sequencing and chipping are widely used in the research of livestock genetic structure, kinship, inbreeding, etc. [5]. And significant progress has been made in this area, which has important reference value for the formulation of livestock breeding and seed conservation strategies. In 2007, reduced-representation genome sequencing (RRGS) technology was proposed by Miller et al. [6]. On the basis of next-generation sequencing (NGS) technology, a method for sequencing specific genomic regions and reflecting the whole genome sequence was proposed [7]. This technology can not only reduce the complexity of the genome but also effectively avoid the disadvantages of the complex process and the high cost of previous high-throughput sequencing methods and improve the reliability of sequencing results without a reference genome. Therefore, it has great advantages in the research of non-reference genome species [8]. For most species, human activities and environmental factors can easily cause changes in gene flow among species, resulting in genetic differentiation of different degrees among populations in the region and changing the genetic structure of species. Therefore, species genomics has become an important area of study in species research [9]. According to the different methods of constructing genome libraries, simplified genome sequencing technology can be divided into RRL (reduced representation library sequencing), RAD (restriction-site associated DNA), and GBS (genotyping by sequencing). GBS uses restriction endonuclease to label genes, and high-density SNP markers of sample species can be obtained through high-throughput parallel sequencing of multiple samples [10]. Compared with RAD technology, the selection of GBS fragments is simpler, there are fewer steps in database construction, and it is more advantageous for species with low polymorphism and high repeat sequences [11]. Although this technology has been widely used, there is no theoretical basis for the Hetian Qing donkey breed group, and research findings on the Hetian Qing donkey are lacking even further, especially at the molecular level. Therefore, this study uses GBS technology to analyze the genetic structure of 32 Hetian Qing donkeys and discusses the degree of genetic diversity, population structure, and differentiation. This is conducive to protecting the resources of Hetian Qing donkeys and has an important significance for the sustainable development of the Xinjiang donkey industry. In view of the above considerations, the purpose of this current study was to analyze the genetic structure of 32 Hetian Qing donkeys by means of the GBS technology and discuss the degree of genetic diversity, population structure, and differentiation of this donkey breed. The knowledge gathered in this investigation could represent an important tool for the protection of the resources of Hetian Qing donkeys and the sustainable development of the Xinjiang donkey industry.

## 2. Materials and Methods

### 2.1. Test Animals

A total of 32 Hetian Qing donkeys were selected from private herdsmen’s homes in Pishan County, Hetan, Xinjiang Province, including 28 females and 4 males. After the selection of the Hetian Qing donkeys, a 5 mL EDTA anticoagulant blood collection vessel was used for jugular vein blood collection. The experimental animals were released after the blood collection was completed, and the blood samples were stored in a −20 °C refrigerator for future use.

### 2.2. GBS Test Flow Method

#### 2.2.1. DNA Extraction

DNA was extracted using the magnetic bead method, and genomic DNA was isolated and purified using the cwe9600 magnetic bead blood DNA kit. After extraction, 2% agarose gel was used to detect the quality, Qubit was used to measure the DNA concentration, and the purity of the DNA sample was detected by a nanodrop spectrophotometer (OD260/OD280 = 1.8–2.0), providing reliable basic data for subsequent experimental research.

#### 2.2.2. Construction of the GBS Library

Based on simplified genome sequencing, the qualified DNA was digested with Mse I restriction endonuclease and a linker with a barcode was added to both sides of the enzyme section. Then, PCR amplification was carried out, and the amplified products were mixed. The required fragments were selected to establish the library. The constructed library was initially quantified using Qubit^®^2.0 and diluted to 1 ng·μL^−1^. The library was examined using an Agilent 2100 Bioanalyzer (Santa Clara, CA, USA). After the length of the inserted fragment was obtained, the effective concentration of the library was accurately quantified using q-PCR (the effective concentration of the library > 2 nmol·L^−1^) to ensure the quality of the library [12]. The library was qualified. The pool was mixed according to the effective concentration of different libraries and the amount of data required for the target machine, followed by Illumina Hi-seq PE150 sequencing [13].

### 2.3. Data Statistics and Analysis

#### 2.3.1. Sequencing Data Quality Control

The raw data of high-throughput sequencing were sorted and preprocessed according to the following steps: Trimmomatic [14] was used to filter the data quality. The filtering conditions were as follows: remove the connector sequence contained in the read; remove the base with a quality of less than 20; filter the filtered data according to the length; remove the reads with a length of less than 50 bp or with only one end; and finally obtain the effective sequencing data.

#### 2.3.2. SNP Detection and Quality Control

The original image data file (Illumina Hi-seq sequencing platform) obtained through sequencing was transformed into an original sequencing sequence, and the effective sequence was obtained after quality control. Samtools [15] were used for preprocessing according to the positioning results of clean reads in the reference genome, such as Mark Duplicates and Base Recalibration. Through the use of the GATK [16], the joint calling method was used to improve the performance of the population analysis. This allowed us to simultaneously detect single nucleotide polymorphisms (SNPs) in multiple samples, filter the samples, and obtain the final SNP locus set, thus ensuring the accuracy of the detected SNP. Plinkv1.90 software was used to perform quality control on the detected SNP data. The following quality control criteria were used for subsequent analysis of the SNP locus genotype data: sequencing depth ≥ 10×; Q20 > 95%; SNP detection rate ≥ 90%; minimum allele frequency (MAF) ≥ 0.01; and a Hardy–Weinberg *p* value ≥ 10^−6^.

#### 2.3.3. Genetic Diversity Analysis

Genetic diversity analysis includes effective population size (Ne), which refers to the ideal population size with the same gene frequency variance or the same inbreeding coefficient increment (heterozygosity decay rate) as the actual population [17]. It is usually estimated based on the linkage disequilibrium (LD) level of the population [18]. Through the use of Plink software, the minimum allele frequency (MAF) [19] of each site was then calculated and analyzed for the proportion of polymorphic markers (PNs) in the population. The expected heterozygosity (He) of the population refers to the probability of heterozygosity of any individual in the population at any site, and the observed heterozygosity (Ho) refers to the proportion of individuals who are heterozygous at a site in the population compared to the total number of individuals. The polymorphism information content (PIC) is an index that measures the degree of gene variation and reflects the amount of genetic information. PIC can be calculated according to the formula of Bostein et al. [20]. The number of effective alleles and the minimum allele frequency (MAF) refer to the frequency of uncommon alleles in a given population.

$$PIC=1-\sum_{i=1}^{n}P_{i}^{2}-\sum_{i=1}^{n-1}\sum_{j=i+1}^{n}2P_{i}^{2}P_{j}^{2}$$

where *P_i_* and *P_j_* are the frequencies of *ith* and *jth* alleles for the selected marker, respectively.

#### 2.3.4. Inbreeding Coefficient Analysis Based on Long Homozygous Fragments

Runs of homozygosity (ROHs) are widely present in all populations. An ROH is a continuous fragment of a homozygous genotype in an individual, which is produced by the complete transmission of homologous haplotypes from the parent to the offspring. The length and frequency of ROH can reflect the group history. A long ROH can indicate a recent genetic relationship; the more such fragments there are, the higher the possibility of inbreeding within the family. A short ROH indicates a genetic relationship that occurred in the distant period. Plink (V1.90) was used to calculate the ROH length of each sample. The coefficient of inbreeding based on ROH is calculated by calculating the proportion of the total length of ROH fragments in the total length of the autosome genome [21]. Therefore, the longer the total length or the higher the number of ROHs in an individual, the higher the inbreeding coefficient of that individual.

FROH = ∑_k_Length(ROH_k_)L

Among them, k is the number of ROHs in an individual, and L is the length of the autosomal genome covered by genotype data (donkey, approximately 2,302,664.694 Kb).

#### 2.3.5. Genetic Relationship Analysis of Population Genome

Principal component analysis (PCA) is a purely mathematical operation method that can select a smaller number of important variables by linearly transforming multiple related variables. PCA is applied for cluster analysis in many disciplines, mainly in genetics. It is based on the degree of individual genomic SNP differences, clustering individuals into different subgroups based on principal components according to different trait characteristics, and is used for mutual validation with other methods. This project used smartpca software based on SNP to conduct principal component analysis (PCA) and obtain the principal component clustering of sample X. Through PCA analysis, it is possible to determine which samples are relatively close and which samples are relatively distant, which can assist in evolutionary analysis. The genomic relationship G matrix was constructed using the genome-wide marker information, and the G matrix molecular kinship analysis was carried out using GCTA (V1.94) software. A heat map was drawn to show the genomic kinship among individuals [22]. Plink (v1.90) software was used to calculate the genetic distance between individuals, construct an identity-by-state (IBS) matrix, and analyze the genetic distance of IBS.

#### 2.3.6. Cluster Analysis to Construct the Hetian Qing Donkey Family

Cluster analysis is a method of generating a relatively simple class structure from a group of complex data and classifying groups according to the degree of correlation or similarity between different individuals [23]. The adjacency method (neighbor-joining, NJ) was used to cluster the samples based on the genetic distance matrix obtained from the genetic distance analysis. Finally, the whole classification system was turned into a genealogy chart, which shows the kinship between all samples and represents the families of the Hetian Qing donkey conservation population.

## 3. Results

### 3.1. Genomic DNA Detection

The concentration of DNA samples from 32 Hetian Qing donkeys was greater than 100 ng/μL. The OD260 nm/OD280 nm ranges from approximately 1.8 to 2.0. The genomic DNA sample was of good quality, had no protein contamination, and had no degradation, thus meeting the analysis requirements. This meant that it could be used for subsequent research.

### 3.2. Sequencing Data Output and Quality Control

According to Table 1, the average effective base number obtained from GBS sequencing and quality control of 32 Hetian green donkey blood genomes was 3,813,442.839. The average GC base content was 41.55%. The average proportion of bases reaching Q20 quality was 96.83%. The average proportion of bases reaching Q30 quality was 91.06%. According to Table 2, it can be seen that the average effective read length obtained was 630,750.875. The average proportion of effective read length comparison to the reference base group was 98.32%. The sequencing depth was 2.47×~3.62×, with an average of 3.17×. This indicates that the quality of GBS sequencing was high, the GC distribution was normal, the sample was not contaminated, and the sequencing was successful. The obtained data met the requirements of subsequent analysis.

### 3.3. SNP Locus Quality Control

**Figure 1:**
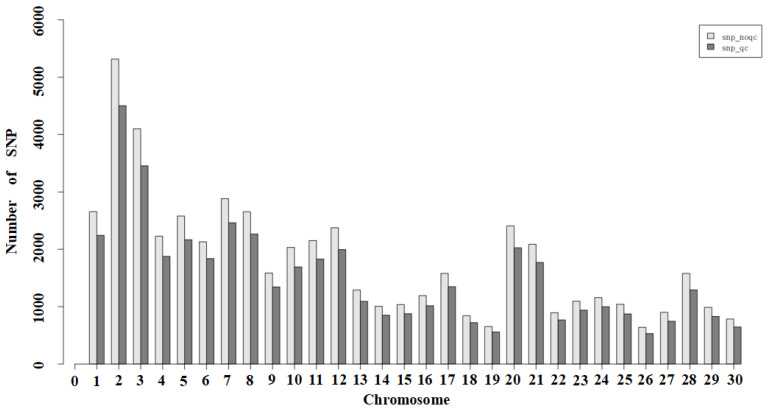
Distribution of SNPs on each chromosome before and after quality control. Note: The X-axis indicates the chromosome number, and the Y-axis indicates the number of SNPs.

Plink (V1.90) software was used to conduct subsequent analysis on the sites with the best SNP typing quality. The specific quality control conditions and results are shown in Table 3. The distribution of SNPs on each chromosome before and after quality control is shown in Figure 1.

### 3.4. Genetic Diversity and Population Structure Analysis

**Figure 2:**
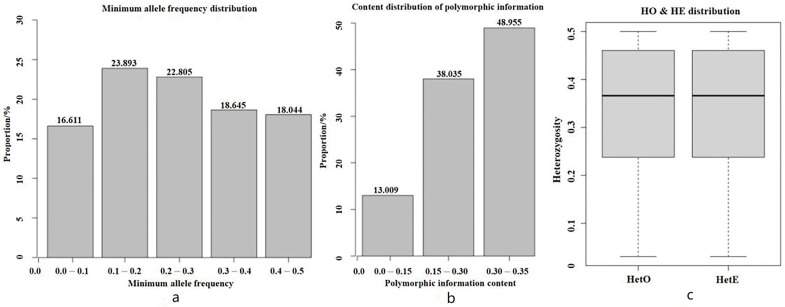
Genetic diversity analysis results. Note: (**a**) Minimum allele frequency distribution (the X-axis indicates the minimum allele frequency interval, and the Y-axis indicates the SNP proportion). (**b**) Distribution of the polymorphism information content (the X-axis represents the PIC interval value, and the Y-axis represents the SNP proportion). (**c**) Heterozygosity analysis (note: the X-axis indicates the classification of Ho and He, and the Y-axis indicates the heterozygosity value).

The results of the genetic diversity analysis are shown in Table 4. A total of 1.536 effective alleles were detected, with an average number of effective alleles of 0.048 and an MAF of 0.250. Among them, there were relatively more between 0.1 and 0.2, accounting for 23.89%. The distribution is shown in Figure 2a. The PN of the SNP locus was 0.955, indicating that 95.5% of the SNP loci were polymorphic, and the polymorphic information content of the SNP locus was 0.273, as shown in Figure 2b. The Ne of Hetian Qing donkeys was 4.1, Ho was 0.347, and He was 0.340. The average observed heterozygosity was slightly higher than the average expected heterozygosity, but the two were very close, as shown in Figure 2c.

### 3.5. Inbreeding Coefficient Analysis Based on Long Homozygous Segments

**Figure 3:**
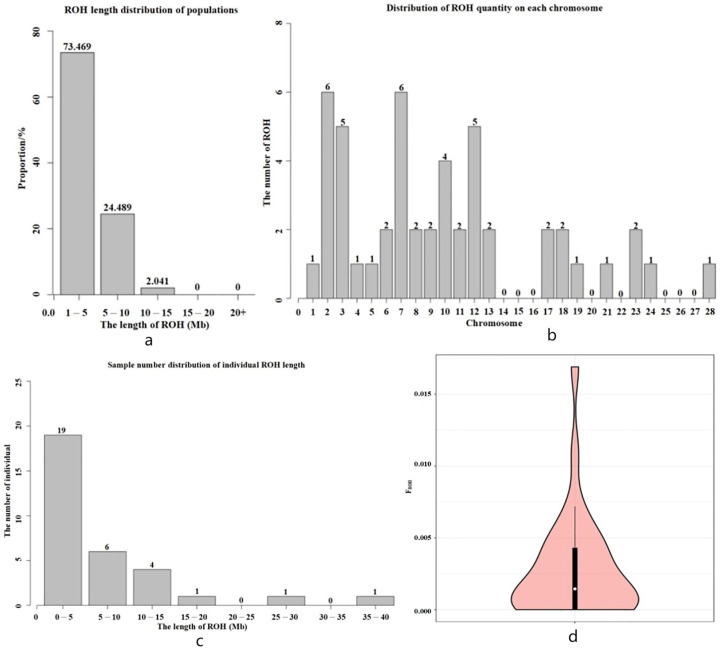
ROH analysis results. Note: (**a**) Distribution of ROH length by population (the X-axis represents the length interval of ROH, and the Y-axis represents the population proportion). (**b**) Distribution of the ROH number on each chromosome (the X-axis represents chromosome number, and the Y-axis represents ROH quantity). (**c**) Sample number distribution of individual ROH length (the X-axis represents the length interval of ROH, and the Y-axis represents the number of individuals). (**d**) The width of the violin chart indicates the probability density distribution of the population’s FROH. The wider part of the violin chart indicates that there are a larger number of samples at this level and vice versa.

As shown in Figure 3a, a total of 49 ROH fragments were detected in the Hetian Qing donkey population. The number of ROH fragments with a length between 1 and 5 Mb was the largest, accounting for 73.46%. The shortest ROH was 1.07 Mb long, located on chromosome 17, and the longest ROH was 10.66 Mb long, located on chromosome 1. As shown in Figure 3b, the number of ROHs on chromosomes 2 and 7 was six, which was the largest. The number of ROHs on chromosomes 14, 15, 16, 20, 22, 25, 26, and 27 was 0. The maximum number of individuals with a total length of ROH between 0 and 5 Mb was 19, as shown in Figure 3c, accounting for 59.37%. The average coefficient of inbreeding of the Hetian Qing donkey population calculated based on ROH was 0.003, most of which was concentrated in 0.00~0.0025, as shown in Figure 3d.

### 3.6. Analysis of Genetic Relationship of Population Genome

#### Kinship Analysis Based on G Matrix

**Figure 4:**
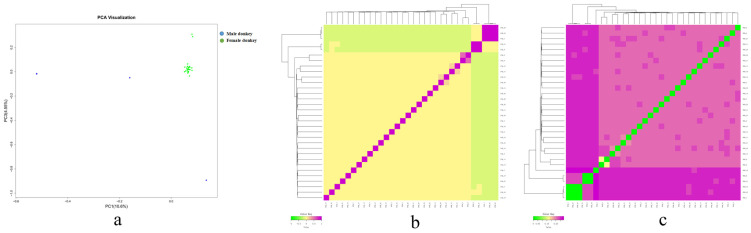
Analysis chart of kinship relationship. Note: (**a**) Principal component analysis results (The X-axis is PC1, and the Y-axis is PC2). (**b**) Visualization results of the genome kinship analysis (The closer the color, the closer the kinship. The X-axis and Y-axis represent the individual IDs). (**c**) Visualization results of the genetic distance analysis (The closer the color is, the closer the genetic relationship is. The X-axis and Y-axis represent the individual IDs).

We performed principal component analysis on 45,557 SNP loci after quality control. From Figure 4a, it can be seen that the Hetian Qing donkeys had a close genetic relationship and high diversity. There were three male donkeys distributed far away. Male and female donkeys were far apart from each other, which clearly indicates diversity. Then, GCTA (V1.94) software was used to calculate the genetic relationship coefficients between individuals, construct a population g matrix (Figure 4b), further analyze the genetic relationship in the protected population of Hetian Qing donkeys, and validate the results of principal component analysis. From Figure 4c, it can be seen that the genetic distance of IBS was 0.011~0.339, with an average of 0.267. Most blocks were relatively light (moderately correlated), and the genetic distance of IBS between most individuals was short, resulting in higher genetic relationships.

### 3.7. Cluster Analysis to Construct the Families in the Hetian Qing Donkey Population

#### Cluster Analysis

**Figure 5:**
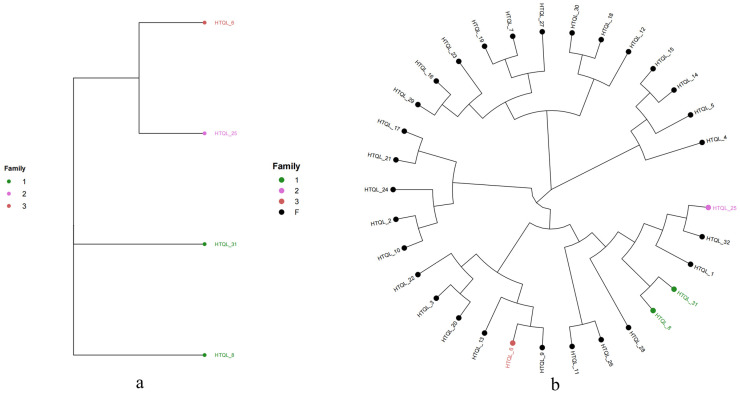
Cluster analysis chart. Note: (**a**) Cluster analysis results of male donkey samples (One color represents a family). (**b**) Cluster analysis results of all samples (The color of the evolutionary tree is the male donkey sample, and one color represents a family).

In view of the importance of the male donkeys to the whole population, we extracted the male donkey samples and conducted cluster analysis separately to judge the distance between them. The results are shown in Figure 5a, and the cluster analysis results of all samples are shown in Figure 5b.

### 3.8. Family Structure Analysis of Hetian Qing Donkey Population

Clustering was conducted based on the genomic phylogenetic relationship and clustering analysis results using genomic phylogenetic coefficients between male donkeys that were greater than or equal to 0.1. The existing male donkey samples could be divided into three families. Different families were divided according to the existing genetic relationship between female donkeys and male donkeys. In addition, 25 female donkeys were found to have distant blood relationships with the tested male donkeys, so they were classified as “other”. The results are shown in Table 5.

## 4. Discussion

### 4.1. Genetic Diversity Analysis

The Hetian Qing donkey is a rare and precious genetic resource in the Hetian region of Xinjiang, China. It plays a vital role in animal husbandry and transportation in southern Xinjiang. With the particularity of its geographical environment, research on the level of genetic diversity and the genetic structure of Hetian Qing donkeys is not only central to protecting the genetic resources of this species but is also the prerequisite for implementing effective scientific variety protection measures [1]. There are many methods of studying the population genetics of species, such as using morphological, cellular, biochemical, and molecular markers. These latter three are vulnerable to environmental and other aspects compared with morphological markers [24]. Previous studies have shown that the limited number of SNPs is the main bottleneck limiting the accuracy of kinship estimation [25]. Therefore, rapid access to a large amount of SNP information is essential to further improve the applicability of molecular markers in estimating genetic relationships. GBS technology can obtain a large number of effective SNPs, provide more genome-level information for molecular markers of species polymorphism, and ensure the accuracy and reliability of the data used for estimating genetic relationships [10,11]. It also has great potential for breeding excellent species when applied with simplified genome sequencing technology to construct genetic maps. Simplified genome sequencing technology can measure genetic data, build phylogenetic trees according to the genetic distance between populations, understand the evolutionary mechanisms and environmental adaptability of a population, and reproduce its evolutionary history, so it has significance in guiding the formulation of conservation strategies and methods [26]. Recently, it has also been widely used in animal genetic diversity analysis, genome characteristics analysis, heterosis evaluation, and other studies [27]. Zhu Wenjin et al. [28] carried out a microsatellite analysis of the genetic diversity and phylogenetic relationship of eight local donkey breeds in China. They showed a highly polymorphic information content of 24 microsatellite loci in eight donkey breeds, except NVHEQ18. The average PIC (0.6940), H (0.7119), and E (3.94) of the eight donkey breeds showed relatively high genetic polymorphism and genetic diversity. Yang Hu et al. [29] carried out a microsatellite genetic analysis of three local donkey breeds in Xinjiang and found that eight microsatellite loci were highly polymorphic in the three populations. The average polymorphic information of three local donkey populations contained PIC (0.7568), and the genetic heterozygosity h (0.7841) was higher than that of other domestic donkey breeds, indicating that the local Xinjiang donkey has rich genetic diversity, a high degree of population genetic variation, and great breeding potential. Through a microsatellite analysis of genetic diversity among Chinese donkey breeds, Zhang RF et al. [30] found that the mean values of PIC, HE, and NE of seven polymorphic loci in 10 donkey breeds were 0.7679, 0.8072, and 6.0275, respectively. In general, Chinese donkeys showed relatively high genetic diversity at the seven polymorphic loci investigated in their study. Furthermore, Lulan Zeng et al. [31] used microsatellite markers to study the genetic diversity in and the genetic relationship of Chinese donkeys. It was found that the average values of polymorphism information content, observed allele number, and expected allele number of all tested Chinese donkeys were 0.6600, 6.890, and 3.700, respectively, indicating that Chinese donkeys have relatively rich genetic diversity. Although there are abundant genetic variations among Chinese donkey breeds, their degree of genetic differentiation is relatively low, accounting for only 5.99% of the total genetic variation among different breeds. Most of the studies on the inbreeding and genetic relationship of local donkey breeds have been based on the microsatellite method, and the application of GBS to study genetic diversity is rare.

The effective population size of the Hetian Qing donkeys measured using GBS in this study was 4.1. Because the expected heterozygosity (He0.340) of the Hetian Qing donkey population was slightly lower than the observed heterozygosity (Ho0.347), it can be considered a moderately heterozygous population. The average observed heterozygosity of the whole population was close to the average expected heterozygosity, which indicates that Hetian Qing donkeys have high purity and rich genetic diversity, which may be more conducive to individual health. Polymorphism information content (PIC) is a good indicator of gene polymorphism. Botstein et al. [20] proposed the index of polymorphism information content to measure the degree of gene variation: when PIC > 0.5, the locus is highly polymorphic; when 0.25 < PIC < 0.5, the locus is moderately polymorphic; and when PIC < 0.25, the locus has a low degree of polymorphism. Their study found that 95.5% of SNPs were polymorphic, with an average PIC of 0.273, representing moderate polymorphism. The PIC value obtained in this study is slightly lower, indicating that there has been a loss of genetic diversity in the process of conservation of Hetian Qing donkeys, and the conservation measures need to be strengthened.

### 4.2. Analysis of Inbreeding Degree

Recently, research on the genomic inbreeding coefficient based on ROH has mainly concentrated on pigs, cattle, and other domestic animals, and many achievements have been made [32]. ROH is usually used to estimate the inbreeding coefficient of a genome. The length of ROH and the proportion of the genome covered by ROH can accurately reflect the age and source of inbred lines, thus reflecting the level of inbreeding. The length of ROH is directly proportional to the genetic relationship between individuals. The longer the ROH segment, the greater the possibility of inbreeding, and vice versa. The more ROH segments there are, the more it is generally believed that a population has inbreeding [33]. A long homozygous segment (ROH) is a continuous homozygous genotype segment existing in individuals in a genome. Inbreeding can improve the homozygosity of populations. The possibility of homozygosity of harmful recessive genes increases with inbreeding, which may reduce the reproduction and survival ability of offspring. Through the detection of ROH fragments in the whole genome of 32 Hetian Qing donkeys, this study found that there were 49 ROH fragments in 32 Hetian Qing donkeys, 73.47% of which were between 1 and 5 Mb long, and the average length of each ROH was 1.75 Mb. The individuals in the Hetian Qing donkey population had the most ROH in the range of 0~5 ROH, and the average inbreeding coefficient was 0.003. The number and length of ROH fragments concisely reflected that the inbreeding degree and artificial selection intensity of Hetian Qing donkeys were not obvious, and seed conservation plays a role. Wang Gang [34] found that the number of ROH and the average ROH length of the Kunsha donkey was the highest (SROH = 17,389.90 Kb) in the study of the genetic structure of the Chinese domestic donkey population through whole-genome sequencing. The average ROH length of the Qinghai donkey was the lowest (SROH = 7746.38 Kb), and the number of ROH was also the lowest. However, the length and number of ROH in the Hetian Qing donkey were lower than those obtained for the Qinghai donkey in Wang Gang’s research results. This may be due to the following two reasons: (1) Xinjiang is a vast area, so it is not easy for Hetian Qing donkeys to inbreed in this area; (2) the Hetian Qing donkeys are neither the only local animal nor the most important economic and transportation animal, so the intensity of artificial selection is relatively low. The low inbreeding degree and low selection pressure may be the factors contributing to the short ROH of the Hetian Qing donkey.

Due to the limitations of the population size and the relatively closed operation mode of the Hetian Qing donkey species in Xinjiang, China, combined with the extension of seed conservation time and the intensification of generation overlap, an increase in the inbreeding coefficient within the population and a change in genetic structure are inevitable trends. In later stages, the breeding plan can be reasonably changed through sequencing data, the breeding method can be altered if necessary, and the stability of the population structure can be guaranteed through artificial insemination and other methods. Therefore, this study provides a valuable reference for the formulation of breeding process selection schemes.

### 4.3. Analysis of Genetic Relationship and Genetic Structure

In this study, 45,557 quality control SNPs were used to construct a G matrix, and the genetic coefficient obtained reflected the true genetic relationship between individuals. Combined with the results of genomic genetic relationship analysis and cluster analysis, the existing male donkey samples could be divided into three families, and the genetic relationship coefficient between male donkeys was greater than or equal to 0.1 as the standard for clustering. The population can be divided into different families according to the genetic relationship between female and male donkeys. In addition, 25 female donkeys were found to be far from the detected male donkeys, so they were classified as “other”. There was a relatively distant genetic relationship between the three male donkeys HTQL_8, HTQL_31, and HTQL_25, which was consistent with the results of the NJ cluster analysis, while the two-dimensional PCA analysis of the 32 Hetian Qing donkeys also showed that there were three points each forming a community that was far from the other communities. The three kinds of analysis based on SNP information in this study obtained largely the same results, which confirmed the authenticity and reliability of the genetic relationship between the 32 Hetian Qing donkeys obtained in this study. 

The average inbreeding coefficient of this population was 0.003. In order to reduce population inbreeding, it is recommended that male and female donkeys of the same family are not bred together. In the results of population family construction, the genetic relationship coefficient of the “other” 25 female donkeys and all male donkeys was less than 0.1, so they can be mated with any male donkey, and then they can be mated after confirming the genetic relationship coefficient. The effective methods to reduce the inbreeding coefficient were to expand the population size, carry out proper selection, and strengthen management to avoid inbreeding caused by incomplete or incorrect pedigree records. This study also found that the male and female donkeys in the conservation population of Hetian Qing donkeys had three families; the number of male and female donkeys in the three families varied greatly, and the family structure was unbalanced. Therefore, it is necessary to introduce new lineages from other regions, expand the core group, implement reasonable selection, avoid inbreeding, and maintain the genetic diversity of the conservation population of the Hetian Qing donkey.

## 5. Conclusions

The 32 Hetian Qing donkeys were divided into six families, with significant differences in individual numbers among each family. The genetic relationships between families can serve as a basis for breeding selection. Family 1, 2, and 3 only have male donkeys (HTQL-8, HTQL-31; HTQL-25; and HTQL-6). During the breeding process, attention should be paid to preventing the loss of diversity among these three families. The inbreeding degree of male donkeys was not high, with an average inbreeding coefficient of 0.003, and there was a large space for random mating. The conservation effect of the population was good and could be compared between generations to further analyze the conservation effect.

## Figures and Tables

**Table 1:** Summary of quality of original sequencing data.

Sample	Raw Reads	Bases	GC (%)	Q20	Q30	Avg. Quality
HTQL.1	3,422,462	0.493	40.550	96.750	90.710	35.430
HTQL.2	2,777,844	0.400	40.515	97.010	91.405	35.550
HTQL.3	3,600,942	0.519	41.420	96.995	91.505	35.560
HTQL.4	3,260,564	0.470	41.700	96.720	90.800	35.435
HTQL.5	3,222,100	0.464	40.960	97.200	91.890	35.635
HTQL.6	3,848,468	0.554	41.705	97.085	91.610	35.585
HTQL.7	3,809,612	0.549	42.090	96.885	91.170	35.505
HTQL.8	3,619,272	0.521	40.420	96.795	90.895	35.460
HTQL.9	3,789,832	0.546	42.050	96.090	89.175	35.155
HTQL.10	3,234,164	0.466	42.210	97.565	92.875	35.805
HTQL.11	3,845,054	0.554	41.370	97.020	91.505	35.565
HTQL.12	4,469,164	0.644	41.740	97.345	92.375	35.715
HTQL.13	4,455,246	0.642	41.955	97.200	92.005	35.645
HTQL.14	4,765,914	0.686	40.885	96.940	91.175	35.510
HTQL.15	3,337,238	0.481	41.090	96.370	89.835	35.270
HTQL.16	3,814,590	0.549	41.725	96.300	89.730	35.250
HTQL.17	3,771,948	0.543	41.920	96.250	89.585	35.225
HTQL.18	3,236,658	0.466	41.120	96.160	89.365	35.185
HTQL.19	3,959,474	0.570	41.800	97.370	92.450	35.725
HTQL.20	3,445,792	0.496	41.320	97.055	91.530	35.570
HTQL.21	4,765,826	0.686	41.810	96.995	91.440	35.550
HTQL.22	4,100,654	0.590	41.680	97.590	92.980	35.820
HTQL.23	3,854,112	0.555	42.180	97.200	91.995	35.645
HTQL.24	3,630,974	0.523	41.470	95.805	88.520	35.035
HTQL.25	4,728,044	0.681	41.710	97.200	91.980	35.645
HTQL.26	4,159,726	0.599	42.155	96.475	90.220	35.335
HTQL.27	3,898,920	0.561	41.670	96.890	91.250	35.515
HTQL.28	3,856,794	0.555	41.740	95.720	88.340	35.000
HTQL.29	4,043,730	0.582	41.905	97.300	92.325	35.705
HTQL.30	3,609,010	0.520	41.720	97.610	93.070	35.830
HTQL.31	3,580,730	0.516	41.330	96.970	91.455	35.550
HTQL.32	3,724,332	0.536	41.690	95.845	88.640	35.055

Note: (1) Sample: sample name; (2) Raw Reads: raw data reads; (3) Bases: data volume, unit: 1G base; (4) GC (%); the proportion of GC in the total alkali base in the original data; (5) Q20: proportion of base mass greater than 20 in original data; (6) Q30: proportion of base mass greater than 30 in original data; (7) Avg. Quality: average quality value.

**Table 2:** Sequencing data quality assessment.

Sample	Clean Reads	Mapped (%)	Properly Mapped (%)	sites_covgMean	sites_numCovg1
HTQL.1	551,791	541,941 (98.21%)	425,834 (77.97%)	0.03	2.84%
HTQL.2	467,850	459,993 (98.32%)	387,516 (83.81%)	0.03	2.47%
HTQL.3	600,936	590,498 (98.26%)	436,264 (73.13%)	0.03	3.07%
HTQL.4	582,825	573,456 (98.39%)	429,726 (74.20%)	0.03	2.96%
HTQL.5	551,144	542,081 (98.36%)	418,744 (76.57%)	0.03	2.85%
HTQL.6	673,348	665,310 (98.81%)	456,548 (68.31%)	0.04	3.40%
HTQL.7	704,081	693,711 (98.53%)	471,428 (67.33%)	0.04	3.49%
HTQL.8	568,191	557,549 (98.13%)	433,046 (77.05%)	0.03	2.92%
HTQL.9	656,737	646,596 (98.46%)	463,620 (71.05%)	0.04	3.25%
HTQL.10	703,473	694,872 (98.78%)	468,196 (66.88%)	0.04	3.52%
HTQL.11	606,125	595,465 (98.24%)	446,142 (74.46%)	0.03	3.06%
HTQL.12	678,126	665,467 (98.13%)	475,464 (70.69%)	0.04	3.38%
HTQL.13	739,489	727,028 (98.31%)	498,570 (67.87%)	0.04	3.62%
HTQL.14	643,864	630,377 (97.91%)	464,144 (73.19%)	0.04	3.25%
HTQL.15	564,200	554,598 (98.30%)	423,004 (75.86%)	0.03	2.89%
HTQL.16	607,283	596,625 (98.24%)	448,570 (74.41%)	0.03	3.07%
HTQL.17	644,551	634,387 (98.42%)	455,880 (71.42%)	0.04	3.20%
HTQL.18	561,027	552,047 (98.40%)	427,840 (76.95%)	0.03	2.89%
HTQL.19	665,494	653,913 (98.26%)	465,114 (70.39%)	0.04	3.33%
HTQL.20	555,928	546,186 (98.25%)	426,748 (77.44%)	0.03	2.87%
HTQL.21	721,117	707,761 (98.15%)	495,622 (69.37%)	0.04	3.51%
HTQL.22	696,444	684,859 (98.34%)	483,484 (69.89%)	0.04	3.43%
HTQL.23	708,215	697,463 (98.48%)	474,366 (67.40%)	0.04	3.50%
HTQL.24	538,091	528,300 (98.18%)	429,102 (80.45%)	0.03	2.77%
HTQL.25	730,915	717,497 (98.16%)	502,852 (69.29%)	0.04	3.59%
HTQL.26	697,874	686,292 (98.34%)	481,554 (69.47%)	0.04	3.45%
HTQL.27	632,039	620,958 (98.25%)	450,908 (71.91%)	0.04	3.18%
HTQL.28	580,896	570,451 (98.20%)	441,650 (76.69%)	0.03	2.95%
HTQL.29	721,030	709,702 (98.43%)	482,280 (67.31%)	0.04	3.55%
HTQL.30	627,885	618,003 (98.43%)	445,624 (71.42%)	0.04	3.21%
HTQL.31	557,792	547,754 (98.20%)	437,112 (78.96%)	0.03	2.90%
HTQL.32	645,267	634,799 (98.38%)	467,584 (72.93%)	0.04	3.21%

Note: (1) Sample: sample name; (2) Clean Reads: the number of filtered clean reads; (3) Mapped (%): number of clean reads mapped to the reference genome; (4) Properly mapped (%): the number of clean reads with both ends of the sequenced sequence located on the reference genome and the distance consistent with the length distribution of the sequenced segment; (5) sites_CovgMean: average coverage depth of all sites in the genome; (6) sites_NumCovg1: the proportion of bases of sequencing depth greater than or equal to 1× on the genome to the total length of the genome.

**Table 3:** Statistical results of SNP quality control.

Quality Control Standard	Number of SNPs
Total number of SNPs	55,399
SNP with MAF < 0.01	3
SNP no tin Hardy–Weinberg equilibrium (*p* < 10^−6^)	318
SNP with callrate < 0.90	7992
SNPs on chromosome X	1434
SNPs on chromosome Y	89
SNPs used after quality control	45,557

**Table 4:** Analysis results of population genetic diversity.

Effective Population Size (Ne)	4.10
Proportion of Polymorphic Markers (PN)	0.955
Expected Heterozygosity (He)	0.340
Observed Heterozygosity (Ho)	0.347
Polymorphism Information Content (PIC)	0.273
Effective Numbers of Alleles	1.536
Minor Allele Frequency (MAF)	0.250

**Table 5:** Results of population and family construction.

Family 1 Male	Family 1 Female	Family 2 Male	Family 2 Female	Family 3 Male	Family 3 Female	Miscellaneous
HTQL_8		HTQL_25	HTQL_32	HTQL_6	HTQL_9	HTQL_30
HTQL_31			HTQL_1			HTQL_4
						HTQL_28
						HTQL_26
						HTQL_16
						HTQL_11
						HTQL_15
						HTQL_12
						HTQL_22
						HTQL_5
						HTQL_3
						HTQL_20
						HTQL_29
						HTQL_19
						HTQL_24
						HTQL_27
						HTQL_2
						HTQL_13
						HTQL_21
						HTQL_23
						HTQL_17
						HTQL_14
						HTQL_10
						HTQL_7
						HTQL_18

## Data Availability

All data generated or analyzed during this study are included in this published article. “The datasets generated and/or analyzed during the current study are available in the [BioProject] repository, [ACCESSION NUMBER: PRJNA1008346]”.
